# Sequential Role of SOXB2 Factors in GABAergic Neuron Specification of the Dorsal Midbrain

**DOI:** 10.3389/fnmol.2018.00152

**Published:** 2018-05-08

**Authors:** Neoklis Makrides, Elena Panayiotou, Pavlos Fanis, Christos Karaiskos, George Lapathitis, Stavros Malas

**Affiliations:** ^1^Developmental and Functional Genetics Group, The Cyprus Institute of Neurology & Genetics and Cyprus School of Molecular Medicine, Nicosia, Cyprus; ^2^Neurologic Clinic A, The Cyprus Institute of Neurology & Genetics, Nicosia, Cyprus; ^3^Department of Molecular Genetics, Function & Therapy, The Cyprus Institute of Neurology & Genetics, Nicosia, Cyprus; ^4^Neuroscience Laboratory, The Cyprus Institute of Neurology & Genetics, Nicosia, Cyprus

**Keywords:** SOX21, SOX14, SOXB2, midbrain, development, GABAergic

## Abstract

Studies proposed a model for embryonic neurogenesis where the expression levels of the SOXB2 and SOXB1 factors regulate the differentiation status of the neural stem cells. However, the precise role of the SOXB2 genes remains controversial. Therefore, this study aims to investigate the effects of individual deletions of the *SOX21* and *SOX14* genes during the development of the dorsal midbrain. We show that SOX21 and SOX14 function distinctly during the commitment of the GABAergic lineage. More explicitly, deletion of *SOX21* reduced the expression of the GABAergic precursor marker GATA3 and BHLHB5 while the expression of GAD6, which marks GABAergic terminal differentiation, was not affected. In contrast deletion of *SOX14* alone was sufficient to inhibit terminal differentiation of the dorsal midbrain GABAergic neurons. Furthermore, we demonstrate through gain-of-function experiments, that despite the homology of *SOX21* and *SOX14*, they have unique gene targets and cannot compensate for the loss of each other. Taken together, these data do not support a pan-neurogenic function for *SOXB2* genes in the dorsal midbrain, but instead they influence, sequentially, the specification of GABAergic neurons.

## Introduction

SOXB factors are divided into two sub-groups according to their transcriptional activity (Uchikawa et al., 1999; Bowles et al., 2000). SOXB1 members (SOX1, SOX2 and SOX3) have been proposed to act as activators while SOXB2 members (SOX14 and SOX21) are thought to act as repressors (Uchikawa et al., 1999). Furthermore, gene expression regulation by the SOX transcription factors is mediated through the formation of complexes with specific partner proteins (Uchikawa et al., 1999; Kamachi et al., 2000, 2001; Kondoh and Kamachi, 2010) as binding of the SOX proteins alone to the promoter elements is not sufficient to mediate a subsequent response (Yuan et al., 1995; Botquin et al., 1998; Kamachi et al., 2000).

SOXB2 factors have been shown to promote neurogenesis, even though this role can only be attributed to SOX21 since SOX14 is not expressed in undifferentiated progenitors (Uchikawa et al., 1999; Sandberg et al., 2005; Cunningham et al., 2008; Wegner, 2011; Delogu et al., 2012; Popovic et al., 2014). *SOX21* has been found to be co-expressed with the *SOXB1* genes in the ventricular zone (VZ) during early neurogenesis (Uchikawa et al., 1999) up until the subventricular zone (SVZ) boundary (Sandberg et al., 2005). Overexpression of *SOX21* in the VZ of chick embryo spinal cords caused a moderate reduction of cell proliferation and concomitant induction of premature differentiation of neural progenitors (Sandberg et al., 2005). However, maintenance of SOX21 expression in committed neuronal progenitors prevented their terminal differentiation (Sandberg et al., 2005). These studies led to the suggestion that the net outcome of antagonistic interactions between SOX21 and SOXB1 factors determines whether a neural progenitor will initiate neuronal differentiation or self-renewal (Bylund et al., 2003; Sandberg et al., 2005; Matsuda et al., 2012). In support of this, studies in human glioma cells demonstrated that induced expression of SOX21 promoted differentiation through direct repression of SOX2, but inhibited neural maturation in mouse PC12 cells (Ohba et al., 2004; Ferletta et al., 2011). Furthermore, adult neurogenesis of the hippocampus in SOX21 deficient mice was shown to be significantly reduced (Matsuda et al., 2012).

Despite these evidence, studies in small cell lung cancers suggest that SOX21 promotes proliferation rather than differentiation (Titulaer et al., 2009) and expression of SOX21 in mouse embryonic stem cells (ESCs) induced the expression of SOX2 and thus maintained their pluripotency (Mallanna et al., 2010; Chakravarthy et al., 2011; Kuzmichev et al., 2012). In addition, a recent study in Xenopus embryos also provided conflicting data to the previous model as overexpression of SOX21 at the two-cell stage embryos was shown to expand the neural plate progenitors and instead repress proneural genes when miss-expressed (Whittington et al., 2015). Furthermore, high levels of SOX21 along with noggin have been shown to induce a 2nd axis formation through activation of SOX2 and SOX3 (Whittington et al., 2015).

Although the SOXB2 genes are evolutionary conserved paralogs, there are limited studies focusing on the specific role of SOX14. *In vivo* studies using SOX14 knockout mice illustrate a defect in the ventral lateral geniculate nucleus GABAergic neuron maturation that originate from the dorsal midbrain (Delogu et al., 2012). Alternatively, the GABAergic neurons of the subcortical visual shell retain their inhibitory fate; possibly due to compensation by SOX21 (Delogu et al., 2012). Furthermore, loss-of-function of SOX14 in chick embryos resulted in the inhibition of GABAergic neuron differentiation of the rostral thalamus (Sellers et al., 2014) indicating that unlike SOX21, SOX14 mediates the terminal differentiation of the neural progenitors rather than their specification.

Drawing from the previously described studies, the function of SOXB2 genes remains controversial and could be both context and region-dependent. Indeed loss-of-function in mice does not support a pan-neurogenic function of SOX21 in all brain regions and there is no obvious evidence for another factor compensating for its loss. In this study we’ve studied the function of SOX21 and SOX14 in the dorsal mid-brain and provide evidence that these factors are both necessary and sufficient to induce GABAergic neuron specification sequentially. This study combines both neuronal differentiation and sub-type specification through the function of SOX21.

## Materials and Methods

### Transgenic Mice

All husbandry and experimental procedures performed on the mice were approved by the Chief of Veterinary services of the Republic of Cyprus (project licence: CY/EXP/PR.L6/2017). The animals used for this study were GATA3^eGFP^ (Panayi et al., 2010), SOX21^KO^ (Kiso et al., 2009), SOX14^KO^ (Crone et al., 2008) and SOX21^GFPn^ generated using the Bacterial Artificial Chromosome (BAC) clone RP23-118F24 and maintained on a C57BL6 background. The mice were kept in a humidity-controlled environment at a constant temperature of 21°C and were subjected to a 12:12 light/dark cycle and with access to water and conventional chow diet (standard Diet: 4RF25 certificate-PF1609, Mucedola, Italy) *ad libitum*. Heterozygote parents were bred together for the SOX21^KO^ and SOX14^KO^ lines to generate knockout offspring. The genotype of the mice was determined through the intensity of the GFP emission and it was confirmed through PCR using the primers listed on Supplementary Table S1.

### Tissue Preparation

Female mice from time-mating crosses were checked daily for the presence of a copulation plug and successful pregnancies were designated as day E0.5, following the finding of the plug. Pregnant mice were sacrificed at the appropriate pregnancy stages through cervical dislocation and the embryos were harvested via cesarean sectioning. The embryonic tissues were fixed in MEMFA (0.1 M MOPS, 2 mM EGTA, 1 mM MgSO_4_, 3.7% paraformaldehyde, pH 7.4) solution for 25–30 min at 4°C depending on the age of the embryo. The tissues were thereafter washed in PBS and cryoprotected in 30% Sucrose solution (in PBS). The samples were placed in cryomolds containing Tissue-Tek^®^ optimal cutting temperature (OCT) compound (Sakura, Japan) and were frozen in pre-cooled isopentane previously stored in −80°C. The tissues were serially sectioned at 6–10 μm thickness in a coronal or sagittal plane using a Bright OTF-5000 Cryostat (Bright Instruments, UK).

### Bromodeoxyuridine (BrdU) Staining

Pregnant mice at the embryonic days of interest were given five intraperitoneal injections within 30 min intervals of BrdU (Sigma, USA) at 50 μg/Kg body weight (dissolved in 0.007 N NaOH in 0.9% NaCl) pre-warmed to 37°C. The mice were sacrificed 2 h following the last injection with BrdU and the embryos were fixed in MEMFA solution. The tissues were sectioned and antigen retrieval was performed with 0.1% TritonX-100 in PBS for 30 min followed by 1 h incubation with 2 M Hydrochloric Acid (HCl) at 37°C. The HCl was neutralized using Hank’s Balanced Salt Solution (HBSS) and the sections were immunostained as previously described (Panayiotou et al., 2013).

### Immunohistochemistry

Immunohistochemistry was performed according to standard procedures using the following primary antibodies: FLAG (fusion protein tag, mouse IgG, Sigma Aldrich, 1:100), SOX14 (guinea pig IgG, Gift from Tom Jessell, 1:2000), GATA3 (lineage-specific marker, rat IgG, gift from Frank Rosvelt, 1:50), DBX1 (lineage-determining factor, rabbit IgG, gift from Alessandra Pieraani, 1:1000), SOX2 (stem cell marker, rabbit IgG, gift from Eumorphia Remboutsika, 1:500), MASH1 (lineage-determining factor, guinea pig IgG, gift from Francois Guillemot, 1:2000), SOX21 (goat IgG, Neuromics USA, 1:200), NKX2.2 (lineage-specific marker, mouse IgG, DSHB USA, 1:50), NKX6.1 (lineage-specific marker, mouse IgG, DSHB USA, 1:50), GAD6 (lineage-specific marker, mouse IgG, DSHB USA, 1:50), GATA2 (lineage-specific marker, mouse IgG, DSHB USA, 1:50), PAX6 (lineage-specific marker, rabbit IgG, Millipore USA, 1:200), BRN3A (lineage-specific marker, goat IgG, Santa Cruz Biotechnology USA, 1:200), LHX2 (lineage-specific marker, goat IgG, Santa Cruz Biotechnology USA, 1:200), BHLHB5 (lineage-specific marker, rabbit IgG, Santa Cruz Biotechnology USA, 1:500), CyclinD1 (proliferation marker, mouse IgG, Santa Cruz Biotechnology USA, 1:50), P27 (cell cycle arrest marker, rabbit IgG, Abcam UK, 1:500), Ki67 (proliferation marker, rabbit IgG, Abcam UK, 1:500) and BrdU (proliferation marker, rat IgG, Abcam UK, 1:100). Alexa Fluor-conjugated secondary antibodies (Thermo Fisher Scientific USA, 1:2000) were used to visualize primary antibody staining and a detailed observation of the slides was conducted using a TCSL confocal microscope (Leica, Germany).

### RT-qPCR

Midbrains were sectioned from E12.5 embryos in chilled RNase free PBS and RNA was isolated using the RNeasy^®^ plus mini kit (Qiagen). RNA integrity and amount were monitored using the OD260/OD280 nm absorption ratio using the NanoDrop 1000 Spectrophotometer (Thermo Fisher Scientific, USA) and 0.5 μg of isolated RNA were reverse transcribed into cDNA using the PrimeScript RT Master Mix (Perfect Real Time; TaKaRa, Japan). The synthesized cDNA was used for Q-PCR amplification using the SsoFast EvaGreen Supermix kit (Bio-Rad Laboratories Inc., USA) and 25 μM of each primer (Supplementary Table S1). Serial dilutions of the cDNA were initially used for Q-PCR amplification of the reference gene GAPDH, in order to evaluate the quality of each cDNA library using the reference gene standard curve. The reliability of the reference gene and the quality of the cDNA libraries was determined by the linear correlation of the threshold crossing point values (Ct) with the logarithmic value of the DNA amount (Pfaffl, 2001). All reactions were performed in technical triplicates and were carried out in a 7900 HT Fast Real-Time RT-PCR System (Applied Biosystems, USA) with an initial activation step at 95°C for 5 s followed by 40 cycles of denaturation at 95°C for 5 s and extension at 60°C for 25 s. The threshold was set at 0.1 fluorescent units and the PCR efficiency and fold induction in comparison to the reference gene GAPDH was calculated according to Pfaffl (2001).

### *In Utero* Electroporation

Plasmids encoding CMV-SOX21-FLAG-IRES-GFP^n^, CMV-SOX14-FLAG-IRES-GFP^n^, CMV-BHLHB5-IRES-GFP^n^, CMV- SOX21HMGpp40VP16-IRES-GFP^n^,        CMV-SOX21HMGpp40 EnR-IRES-GFP^n^ CMV-SOX1HMGpp40VP16-IRES-GFP^n^ and CMV-SOX1HMGpp40EnR-IRES-GFP^n^ were used for gain-of-function experiments. Pregnant mice at the embryonic days of interest were anesthetized using Tribromoethanol (Avertin) administered via intraperitoneal injection at 250 mg/Kg. The uterine horns were exposed through an incision at the abdominal midline and 1 µg/μl of plasmid DNA was injected using a glass micropipette in the embryos mesocoel as described previously (Matsui et al., 2011). Round plate platinum electrodes 5 mm (Nepa Gene, Japan) were placed outside the uterus on either side of the embryo mesencephalon and 5 pulses of 25–30 mV (50 ms-on and 950 ms-off) were applied depending on the embryonic age using a TSS20 OVODYNE Electroporator (INTRACEL, UK). The incision was surgically sutured according to establish protocols (Matsui et al., 2011) and the embryos were harvested 24 h following the electroporation.

### CLARITY

A modified tissue preparation protocol for CLARITY hydrogel mesh stabilization was used. Mouse embryos were harvested at the embryonic days of interest as described previously and immersed in the hydrogel mixture (3, 7% MEMFA, 4% acrylamide (Bio-Rad, USA), 0.05% bis-acrylamide (Bio-Rad Laboratories Inc., USA), 0.25% VA044 Initiator (Wako Chemicals Inc., USA)) at 4°C for 2–3 days. Following the incubation, the mixture was allowed to polymerize at 37°C for 3 h. The embryos were separated from the gel and submerged in clearing solution pH 8.5 (200 mM Boric acid, 4% SDS and PBS) at 50°C until the tissues became transparent. The tissues were then submerged in 80% glycerol and were visualized under a TCSL confocal microscope.

### Lentiviral Vector Production and Delivery

Lentiviral CMV-GFP (pLenti CMV GFP Puro) and shRNA vectors targeting SOX21 (PLKO.1-shSOX21), SOX14 (PLKO.1-shSOX14) and BHLHB5 (PLKO.1-shBHLHB5) were constructed according to the protocol of PLKO.1-puro vector (Addgene, USA) using the oligos in Supplementary Table S1. Retroviruses were produced by transient transfection of HEK293T cells with pMD2_VSVG, psPAX6 and the transfer vector using polyethyliminine (at a ratio of PEI:pVSVG:psPAX2:PLKO.1 = 24:1:3:4) according to manufacturer’s instructions (Sigma-Aldrich, Germany). The medium was removed 4 h following transfection and the supernatants were harvested three times at a 24 h interval. Viral particles were concentrated by centrifugation at 20,000 rpm for 4 h at 4°C and the pellet was resuspended in PBS. The viral infection of E8.5 embryos was accomplished through intra-amniotic injections with 2 μl of the viral vector as described previously (Stitelman et al., 2010).

### Statistical Analysis

For the immunohistochemistry experiments, the number of nuclei and the percentage of the area of expression of every third section per embryo that correspond to 4–6 sections per data set were quantified using the freeware ImageJ. In regards to nuclear expressions, the numbers of nuclei were counted only in the dorsal midbrain which included cells dorsal to the ventrolateral midbrain sulcus. In contrast, cytoplasmic expressions such as GAD6 were measured in the entire midbrain since individual cells could not have been distinguished. In the case of the Gata3/GFP positive cells, quantification was based on the endogenous expression of GATA3 in the GATA3^eGFP^/SOX21^+/−^ due to saturated GFP expression intensity. In the knockout (GATA3^eGFP^/SOX21^−/−^) however reduced GFP intensity allowed for direct cell quantification. The mean values of the previous quantifications were used to distinguish differences between wild type and mutant embryos using the Mann-Whitney U Test (non-parametric) due to the small sample size of each group since each experiment was repeated five times and therefore could not determine the distribution of scores of each group. For the real time Q-PCR experiments, the Ct values were adjusted according to Pfaffl (2001). The adjusted values were then used to determine any statistical differences using Kruskal Wallis test due to the small sample size. The survival of the Sox21^−/−^ mice in comparison to the wild type was analyzed using the test of proportions since there was no lethality in the wild type group.

## Results

### Expression of SOXB2 Genes in the Developing Midbrain

**Figure 1:**
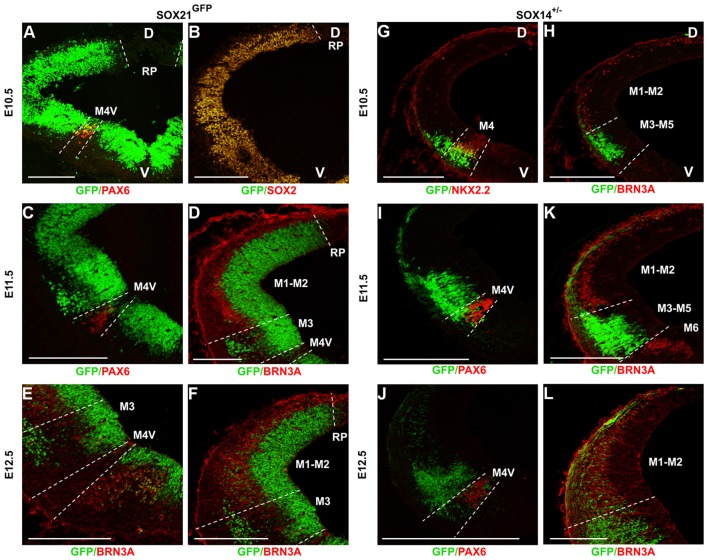
Expression of the SOXB2 genes during early midbrain development. Midbrain coronal sections of *SOX21^GFPn^* midbrains were immunostained at E10.5 **(A,B)**, E11.5 **(C,D)** and E12.5 **(E,F)** for the determination of the *SOX21* expression during neurogenesis. Staining of E10.5 embryos with PAX6 **(A)** and SOX2 **(B)** illustrated that *SOX21* is initially expressed throughout the ventricular zone (VZ) excluding the M4-V domain. At E11.5 a ventral population of SOX21^+ve^ cells emerges from the M3 domain of the VZ as shown by the expression of PAX6 **(C)** and BRN3A **(D)**. At E12.5 the SOX21^+ve^ cell population expands at the dorsal mantle zone (MZ) **(E,F)**. The expression of SOX14 was also mapped using midbrain coronal sections of *SOX14*^+/−^ embryos at E10.5 **(G,H)**, E11.5 **(I,K)** and E12.5 **(J,L)**. At E10.5 and E11.5 a ventral population of SOX14^+ve^ neurons emerges from the M3 and M4 domains of the VZ as shown by the expression of NKX2.2 **(G)**, PAX6 **(I)** and BRN3A **(H,K)**. At E12.5 the SOX14^+ve^ cell population expands at the dorsal MZ **(J,L)**. D—dorsal, V—ventral, Scale bar, 150 µm.

To analyze the expression of the SOXB2 genes in the developing midbrain, we used both antibody staining and two reporter lines, SOX21^GFPn^ (BAC transgenic) and the SOX14^+/−^ (“knock-in” line; Crone et al., 2008). The fidelity of transgene expression relative to the endogenous gene was initially validated by antibody staining (Supplementary Figure S1). At E10.5, SOX21 was detected in the VZ of the developing midbrain, as indicated by the co-localization of the GFP with the VZ marker SOX2, excluding the roof plate and at reduced levels at the M4-V region that is characterized by the expression of PAX6 (Waite et al., 2011; Figures 1A,B). At E11.5, the expression of SOX21 in the VZ remains unaltered and a ventral population of SOX21^+ve^ cells emerges in the mantle zone (MZ). To further map the expression of the ventral population of cells the sections were stained with PAX6 and BRN3A antibodies. In the ventral midbrain, PAX6 marks the M4-V post-mitotic cells while BRN3A is expressed both in the ventral M6 post-mitotic cells progenitors and the dorsal midbrain M1-M2 post-mitotic cells (Nakatani et al., 2007). The SOX21^+ve^ cells were shown to emerge from the M3 progenitor domain and migrate toward the MZ, as indicated by the borders of PAX6 and BRN3A expression (Figures 1C,D). At E12.5, this ventral population of SOX21^+ve^ cells is expanded dorsally and SOX21^+ve^ neurons emerged in a mosaic manner at the dorsal MZ (M1 and M2; Figures 1E,F).

Unlike SOX21, which is also expressed in the VZ progenitors, SOX14 marked exclusively post-mitotic cells of the developing midbrain. At E10.5, SOX14^+ve^ cells emerged from the M4 and M3 domains of the midbrain as delineated by co-staining with NKX2.2 which marks the M4 neurons and BRN3A (Figures 1G,H). At E11.5, the population of SOX14^+ve^ neurons remained at the M4 and M3 domains as shown by the PAX6 and BRN3A staining (Figures 1I,K) and at later stages, the expression of SOX14 expanded throughout the dorsal MZ in a mosaic manner (Figures 1J,L).

### SOXB2 Positive Cells Mark the GABAergic Neurons of the Dorsal Midbrain

In the scope of this study, we focused on the role of the SOXB2 genes during the development of the dorsal midbrain (M1 and M2) at E12.5 when the SOXB2 positive post mitotic neurons emerge. Therefore, we compared their pattern of expression using the SOX21^GFPn^ and the SOX14^KI^ mouse lines to several lineage-specific markers of the M1 and M2 according to the dorsal-ventral gene expression maps published by Nakatani et al. (2007), Waite et al. (2011) and Achim et al. (2013).

**Figure 2:**
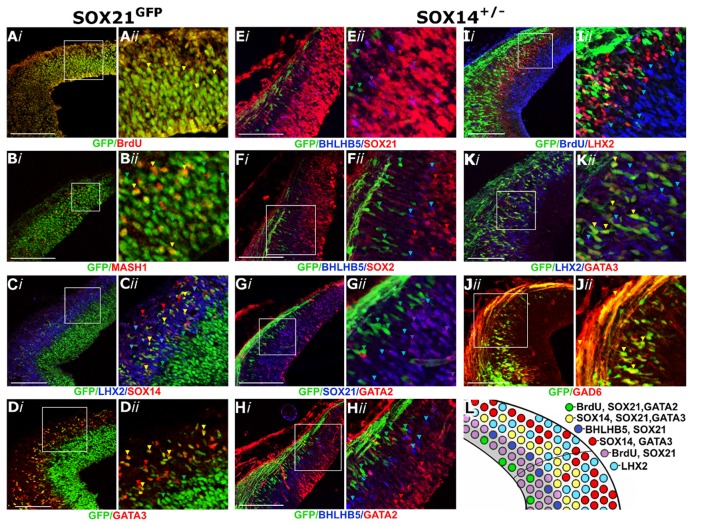
Characterization of the SOXB2 expression in the developing dorsal midbrain at E12.5. Coronal sections of *SOX21^GFPn^* midbrains stained with BrdU **(A)**, MASH1 **(B)**, LHX2 and SOX14 **(C)** and GATA3 **(D)** antibodies. Coronal sections of *SOX14^+/−^* midbrains were stained with BHLHB5 and SOX21 **(E)**, BHLHB5 and SOX2 **(F)**, SOX21 and GATA2 **(G)**, BHLHB5 and GATA2 **(H)**, BrdU and LHX2 **(I)**, GATA3 and LHX2 **(K)** and GAD6 **(J)** antibodies. The results of this analysis are summarized in the schematic **(L)**. Red, green and blue arrows indicate the corresponding single stained cells (as shown in the label) and yellow and purple arrows denote co-localization between green and red cells and blue and red, respectively. Scale bar, 150 μm.

Since SOX21 was detected in the VZ, we examined whether its expression was restricted to either the neural progenitors or the neuronal precursors. Interestingly, SOX21 was shown to be ubiquitously expressed in the VZ as it co-localized with both the BrdU^+ve^ proliferating progenitors and the post mitotic lineage-determining factor MASH1, known to play a role in specifying GABAergic neurons (Wende et al., 2015; Figures 2A,B). Furthermore, we examined the lineage of the SOX21^+ve^ neurons in the MZ of the dorsal midbrain. Immunostaining of the SOX21^GFPn^ sections with the terminal differentiation markers for the glutamatergic and GABAergic lineages, LHX2 and GATA3 respectively, revealed that SOX21^+ve^ cells belong to a subset of GABAergic neurons due to the partial co-localization with GATA3 (Figures 2C,D). Furthermore, triple staining of SOX21^GFPn^, LHX2 and SOX14 revealed that all SOX21^+ve^ post-mitotic cells belong to a subset of SOX14^+ve^ cells which are mutually exclusive with LHX2 (Figure 2C).

Despite the partial co-localization of SOX21 and SOX14 in the MZ, Staining of the SOX14^KI^ sections with the SOX21 antibody revealed a clear boundary of expression between the SOX21^+ve^ progenitors and the SOX14^+ve^ neurons (Figure 2E). Furthermore, BHLHB5, an unknown lineage marker in the dorsal midbrain, was co-localized with SOX21 at the borders of the VZ (Figure 2E), presumably marking the SVZ of the developing midbrain since BHLHB5^+ve^ cells did not express the VZ marker SOX2 (Figure 2F). Similarly to the SOX21 staining, there was a clear boundary of expression between both BHLHB5 and SOX14/GATA3 (Figures 2E,F, Supplementary S2A) demonstrating that SOX14 is only expressed in the MZ. We next examined whether GATA2 was co-expressed with either SOX21 or SOX14 since it has been shown to be expressed both in the VZ and MZ at E12.5 (Kala et al., 2009). GATA2 was indeed found to be co-localized with SOX21 at the VZ (Figure 2G) but was not co-expressed with BHLHB5 or SOX14 (Figures 2G,H).

SOX14 exclusively expressed in the MZ (Figures 2E–G,I) was shown to completely co-localize with all the GATA3^+ve^ GABAergic neurons (Figure 2K) but not the LHX2^+ve^ glutamatergic neurons (Figures 2I,K). In addition, the expression of eGFP in the SOX14^+ve^ neurons allowed for the visualization of their axons which extend dorsally at the roof plate and expressed GAD6 (Figure 2J). Taken together these data show that the SOXB2 genes are expressed in the GABAergic neurons of the dorsal midbrain as shown in the illustration depicting the expression profile of the dorsal midbrain (Figure 2L).

### SOX21 Is Required for the Maintenance of GABAergic Neurons in the Dorsal Midbrain

**Figure 3:**
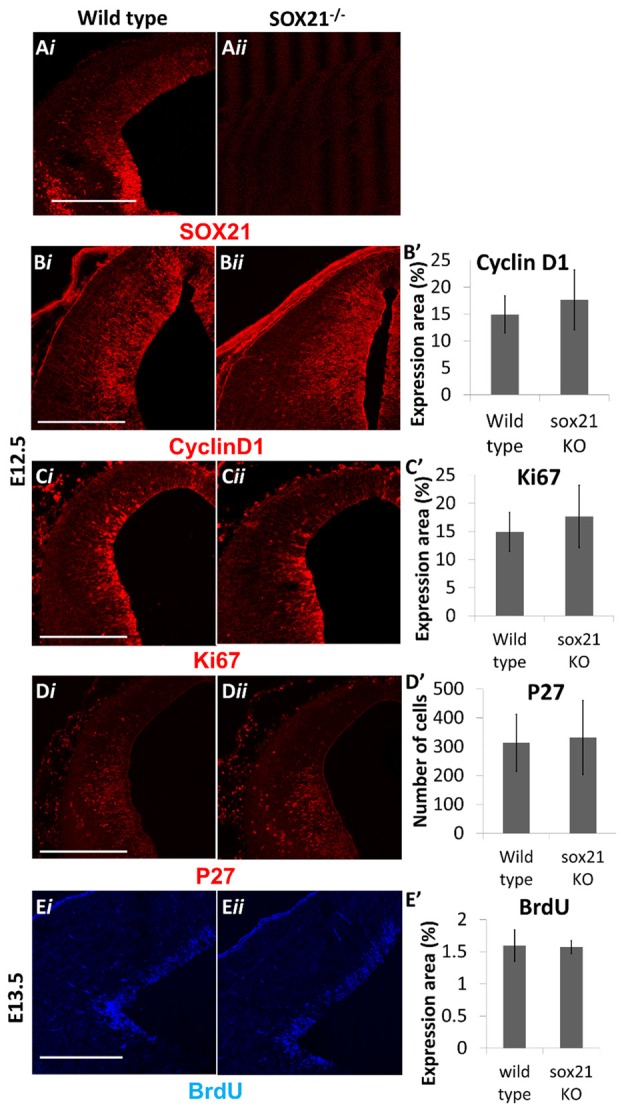
Analysis of cell proliferation in SOX21^−/−^ embryos. The absence of SOX21 in the knockout embryos was validated by antibody staining **(A)**. The area of expression of the cell cycle markers CyclinD1 **(B,B′)**, Ki67 **(C,C′)** and BrdU **(E,E′)** by antibody staining was similar between knockout (*ii*) and wild type embryos (*i*) as well as no differences between the numbers of cell cycle arrest p27^+ve^ cells were observed **(D,D′)**. Data are represented as mean ± SEM, *n* = 10 (5 pairs of mice). Scale bar, 150 μm.

**Figure 4:**
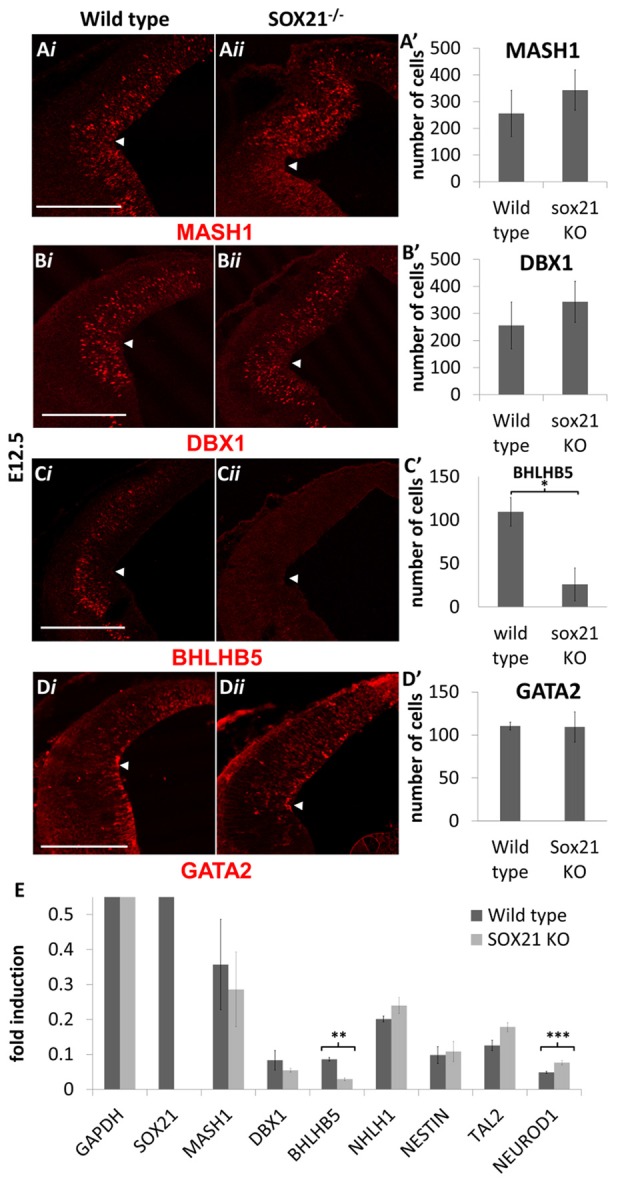
Analysis of lineage-determining factors in SOX21^−/−^ embryos at E12.5. The immunostaining of the lineage-determining factors MASH1 **(A,A′)**, GATA2 **(B,B′)** and DBX1 **(C,C′)** was similar between knockout (*ii*) and wild type embryos (*i*). A significant reduction in the BHLHB5 staining was observed in the Knockout embryos in comparison to wild type **(D,D′)**. All nuclei were counted dorsally to the ventrolateral midbrain sulcus (indicated by white arrow). These data were confirmed by RT-Q-PCR analysis of embryos at the same age **(E)**. Data are represented as mean ± SEM, *n* = 10 (5 pairs of mice). *n = 10 (5 pairs of mice), *P* = 0.0116, two-tailed Mann-Whitney test. **n = 6 (3 pairs of mice), *p* = 0.025, *DF* = 1, Kruskal Wallis test. ***n = 6 (3 pairs of mice), *p* = 0.0253, *DF* = 1, Kruskal Wallis test. Scale bar, 150 μm.

SOX21^−/−^ mice were generated to determine the role of SOX21 in the development of the dorsal midbrain. These mice present with severe loss of skin hair (Kiso et al., 2009), were infertile and approximately 70% expire within the first month (Supplementary Figure S3). Knockout embryos were genotyped though standard polymerase chain reaction (PCR; Supplementary Table S1) and the absence of SOX21 in these embryos was confirmed via antibody staining (Figure 3A). Since SOX21 is expressed throughout the VZ and SVZ of the M1 and M2 domains, we quantitatively compared the expression of cell-cycle markers including CyclinD1, Ki67 and BrdU-incorporation between wild type and mutant embryos in the dorsal midbrain and found no significant differences in the expression of these markers (Figures 3B,B′,C,C′,E,E′). Furthermore, we investigated whether the absence of SOX21 caused any dysregulation of cell cycle arrest or premature differentiation but found no significant differences between wild type and mutant embryos when staining with the cell cycle inhibitor P27 (Figures 3D,D′ and the post-mitotic neuronal marker TUJ1 (Supplementary Figure S4). These data support the notion that loss of SOX21 alone does not affect cell proliferation in the dorsal midbrain during early development. Furthermore, we analyzed the expression of the lineage-determining factors MASH1/GATA2 and DBX1. No significant differences between mutant and wild type embryos were observed when analyzed both with immunostaining and Reverse Transcription Quantitative Real-Time PCR (RT-qPCR; Figures 4A,A′,B,B′,C,C′,E). Despite no noticeable changes in the process of neurogenesis and in the initial lineage specification of GABAergic and glutamatergic neurons, BHLHB5 was dramatically reduced in the absence of SOX21 (Figures 4D,D′,E) while NEUROD1, a negative regulator of BHLHB5 (Peyton et al., 1996), was found to be significantly increased in the RT-qPCR experiments (Figure 4E).

**Figure 5:**
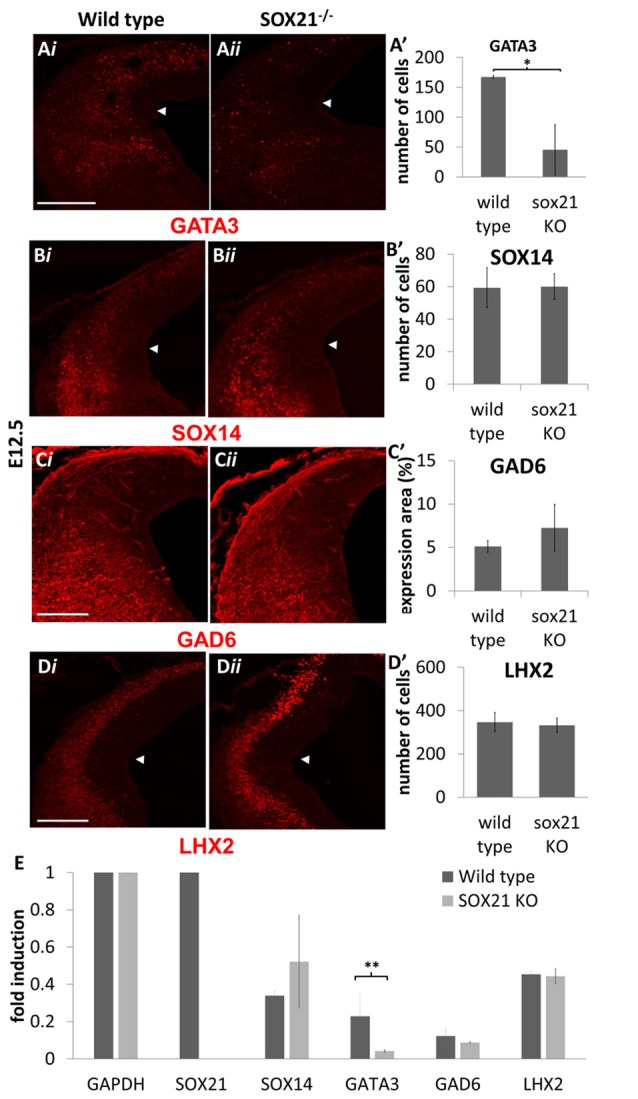
Analysis of the GABAergic and glutamatergic lineages in SOX21^−/−^ embryos at E12.5. The immunostaining of the GABAergic marker GATA3 **(A,A’)** was shown to be reduced in the knockout embryos (*ii*) in comparison to the wild type (*i*). However, the expression of SOX14 **(B,B’)** and GAD6 **(C,C’)** as well as the glutamatergic marker LHX2 **(D,D’)** were not affected in the absence of SOX21. All nuclei were counted dorsally to the ventrolateral midbrain sulcus (indicated by white arrow). These data were confirmed by RT-Q-PCR analysis of embryos at the same age **(E)**. Data are represented as mean ± SEM. *n = 10 (5 pairs of mice), *P* = 0.006, two-tailed Mann-Whitney test. **n = 6 (3 pairs of mice), *p* = 0.042, *DF* = 1, Kruskal Wallis test. Scale bar, 150 μm.

**Figure 6:**
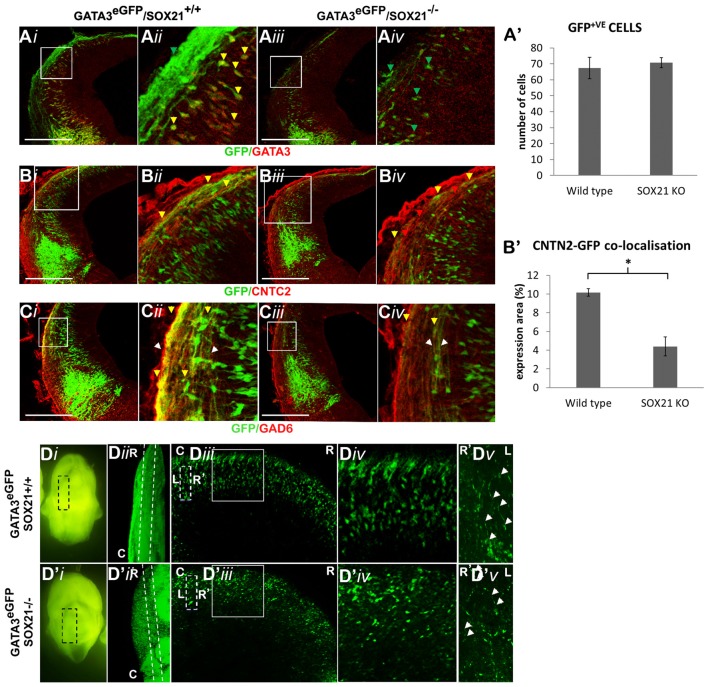
Analysis of the GABAergic neuron maturation in GATA3^eGFP^/SOX21^−/−^ E12.5 embryos. The immunostaining of the GABAergic marker GATA3 was shown to be reduced in the knockout embryos (*iii,iv*) in comparison to the wild type (*i,ii*) despite no noticeable changes in the expression of GFP **(A,A′)**. Immunostaining of the sections with CNTN2 illustrated a significant reduction in the co-localization with the GFP^+ve^ axons in the absence of SOX21 **(B,B′)** as well as a decrease in the thickness of GAD6^+ve^ axons **(C)**—white arrows denote the thickness of the axonal bundle, yellow arrows denote GAD6 co-localization.In addition, the arrangement of the GFP^+ve^ cells was investigated in E12.5 clarified midbrains **(D)**.The dashed lines of the panel (**D***iii*,**D′***iii*) denote the magnified region shown in panel (**D***v*,**D′***v*) and the white arrows indicate the axonal length (R-Rostral, C-Caudal, L-Left, R-Right).Data are represented as mean ± SEM. *n = 10 (5 pairs of mice), *P* = 0.012, two-tailed Mann-Whitney test. Scale bar, 150 μm.

We then examined the expression of the MZ markers and noted that in the mutant embryos there was a two-fold reduction in the expression of GATA3 (Figures 5A,A′). Interestingly, the expression of SOX14 and GAD6 remained unaffected in the absence of SOX21 in spite of the dramatic reduction of GATA3 (Figures 5B,B′,C,C′). Additionally, no abnormalities in the expression of LHX2 were detected (Figures 5D,D′). These findings were consistent with our RT-qPCR results (Figure 5E). However, it remained unclear whether the reduction of GATA3 is due to lack of induction or due to failure to maintain its expression. To investigate this, we crossed GATA3^eGFP^/SOX21^+/−^ compound heterozygous mice to generate SOX21 knockout embryos which carry an eGFP reporter for GATA3. Since eGFP has a long half-life (Corish and Tyler-Smith, 1999), we aimed to use its expression as a reporter of how many GATA3 neurons are generated in both wild type and knockout embryos. Despite the reduction of GATA3 in the SOX21 knockout embryos, there was no significant difference in the numbers of the GFP^+ve^ cells even though a reduction in the density of the GFP^+ve^ axons was detected (Figures 6A,A′). To confirm this finding GATA3^eGFP^/SOX21^−/−^ midbrains were stained with the axon growth marker CNTN2 that was previously found to colocalize with the GFP^+ve^ axons of the dorsal midbrain (Supplementary Figure S2B). Interestingly, in the absence of SOX21 there was a significant reduction in the co-localization of the GFP^+ve^ axons with CNTN2 (Figures 6B,B′). Moreover, staining with GAD6 demonstrated that while there was complete colocalization with the GFP^+ve^ axons in the knockout embryos, the GABAergic axons appeared sparser (Figure 6C). It is unclear however if this phenotype was due to disorganization of the GABAergic neurons in the absence of SOX21. To investigate this further, clarified E12.5 brains from GATA3^eGFP^/SOX21^−/−^ mice were used to visualize the arrangement of the GATA3 positive neurons in the dorsal midbrain using the GFP reporter. Interestingly, mice lacking SOX21 again exhibited shorter axons in comparison to wild type embryos despite no noticeable abnormalities in the density and topology of the neurons (Figure 6D).

### SOX14 Is Required for the Terminal Differentiation of the GABAergic Neurons in the Dorsal Midbrain

**Figure 7:**
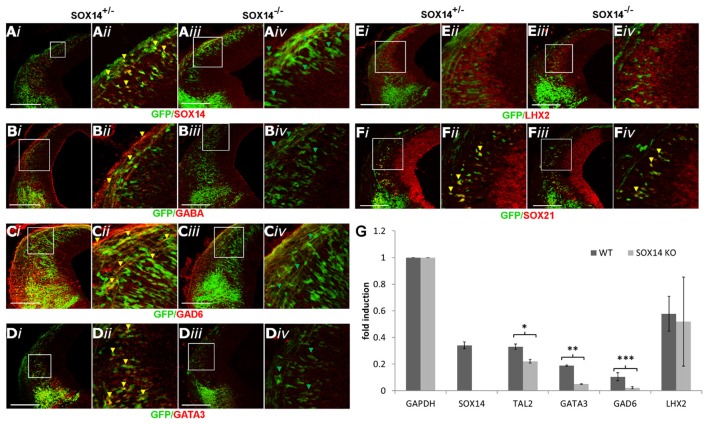
Analysis of the GABAergic and glutamatergic lineages in SOX14^−/−^ embryos at E12.5. The absence of SOX14 in the knockout embryos was validated by antibody staining **(A)**.The immunostaining of the GABAergic markers GABA, GAD6 and GATA3 **(B,C,E)** were shown to be reduced in the knockout embryos (*iii,iv*) in comparison to the heterozygotes (*i,ii*). However, the expression of SOX21 **(D)** and the glutamatergic marker LHX2 **(F)** were not affected in the absence of SOX14. These data were confirmed by RT-Q-PCR analysis of embryos at the same age **(G)**. Data are represented as mean ± SEM. *n = 6 (3 pairs of mice), *P* = 0.032, *DF* = 1, Kruskal Wallis test. **n = 6 (3 pairs of mice), *P* = 0.034, *DF* = 1, Kruskal Wallis test. ***n = 6 (3 pairs of mice), *p* = 0.021, *DF* = 1, Kruskal Wallis test. Scale bar, 150 μm.

Although loss of SOX21 resulted in the reduction of GATA3, it was not sufficient to impair GABAergic terminal differentiation, speculating that SOX14 may indeed compensate for the loss of SOX21. To test this hypothesis, we generated SOX14^−/−^ embryos through crosses of heterozygote SOX14^KI^ parents. Knockout embryos were genotyped though PCR (Supplementary Table S1) and the absence of SOX14 in these embryos was confirmed by antibody staining (Figure 7A). SOX14^−/−^ mice exhibited normal development, the females were fertile, did not die prematurely but were characterized by severe atonia. Embryos at E12.5 lacking SOX14, appeared to have a severe loss of all the GABAergic markers including GABA, GAD6 and GATA3 (Figures 7B–D,G) while the glutamatergic marker LHX2 was unaffected (Figures 7E,G). In addition, TAL2, a terminal selector along with GATA2 for the GABAergic neuron differentiation (Achim et al., 2013), was found to be reduced in the RT-qPCR experiments (Figure 7G). The expression of SOX21 was not affected by the loss of SOX14 (Figures 7F,G), suggesting that the production and expression of the SOXB2 genes is not regulated by each other. The fact that glutamatergic neuron marker expression remained unaltered in SOX14 knockout mice indicates that the loss of GABAergic marker expression is most likely caused by a differentiation defect rather than a fate switch.

### Distinct Role of the SOXB2 Genes During GABAergic Differentiation

**Figure 8:**
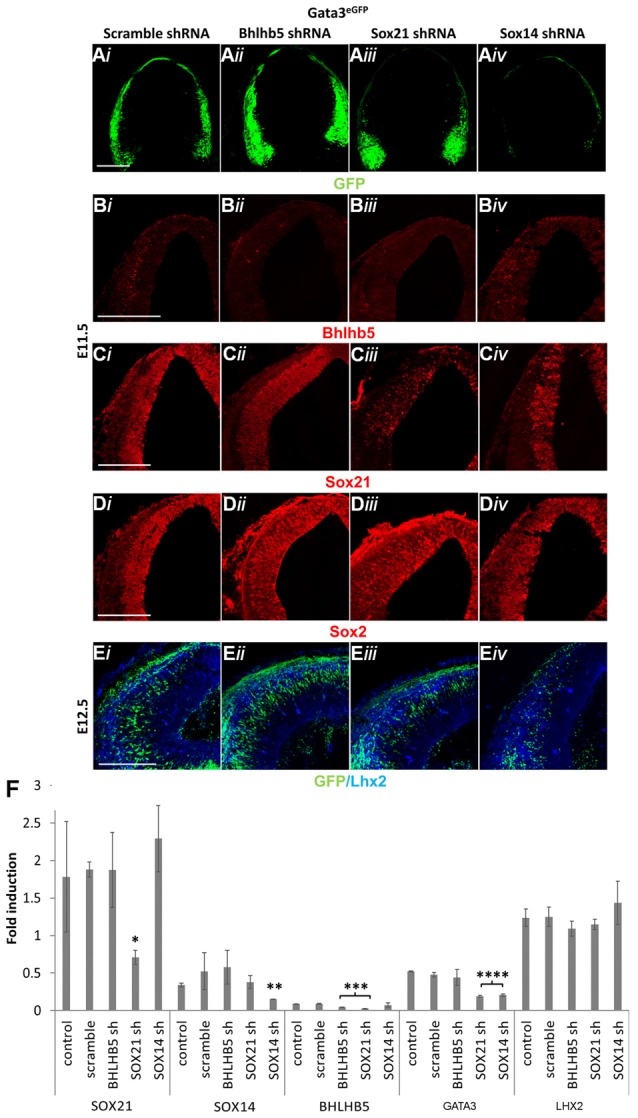
Lentiviral knock down of SOX21, SOX14 and BHLHB5. GATA3^eGFP^ embryos were infected through intra amniotic injections at E8.5 with lentiviral shRNA vectors targeting BHLHB5 (PLKO.1-shBHLHB5; *ii*), SOX21 (PLKO.1-shSOX21; *iii*) and SOX14 (PLKO.1-shSOX14; *iv*). The expression of GFP was reduced in the embryos infected with the SOX14 shRNA vector **(A,E)**. Immunostaining of the embryos with BHLHB5 showed a reduction when embryos were infected with PLKO.1-shBHLHB5 and PLKO.1-shSOX21 **(B)**. Not surprisingly, SOX21 was only reduced when embryos were infected with PLKO.1-shSOX21 **(C)** while not noticeable changes were observed in the expression of SOX2 **(D)** and LHX2 **(E)** when infected with either vector. These data were confirmed by RT-Q-PCR analysis of embryos at E12.5 **(F)**. Data are represented as mean ± SEM. *n = 6 (3 pairs of mice), SOX21sh *p* = 0.0417, *DF* = 4, Kruskal Wallis test, **n = 6 (3 pairs of mice), SOX14sh *p* = 0.0141, *DF* = 4, Kruskal Wallis test, ***n = 6 (3 pairs of mice), SOX21sh *p* = 0.016, BHLHB5sh *p* = 0.0474, *DF* = 4, Kruskal Wallis test, ****n = 6 (3 pairs of mice), SOX21sh *p* = 0.0064, SOX14sh *p* = 0.0021, *DF* = 4, Kruskal Wallis test. Scale bar, 150 μm.

Our results in this study so far indicate that the absence of either of the SOXB2 factors causes a dramatic reduction in the expression of GATA3. However, it is unclear whether the loss of GATA3 in the SOX14 knock out mice was due to lack of induction or due to failure of maintaining its expression in a similar manner as SOX21. Furthermore, the effect of BHLHB5 reduction in the SOX21 knockout mice in the generation of both GABAergic and glutamatergic neurons is not determined. To investigate these, lentiviral vectors were used to knock down the expression of BHLHB5, SOX21 and SOX14 in GATA3^eGFP^ embryos. Unlike with BHLHB5 and SOX21, knock down of SOX14 caused a dramatic reduction of GFP, indicating that the lack of GATA3 in SOX14 knock out mice was due to failure of inducing its expression (Figures 8A,F). In addition, lentiviral knock down of SOX14 did not cause any reduction of BHLHB5 in contrast with the knock down of SOX21 and BHLHB5 (Figures 8B,F). This finding was not surprising since despite the homology of the SOXB2 genes, the SOX21 and SOX14 shRNA seemed to be specific to their targets as shown by staining with SOX21 and SOX2 as well as in the RT-qPCR results (Figures 8C,D,F). Finally, lentiviral knock down of BHLHB5 did not cause any obvious reduction in the expression of GATA3 or LHX2 (Figures 8E,F) confirming that the reduction of GATA3 in the SOX21 knock out and knock down experiments was not associated with the reduction of BHLHB5.

### SOX21 and SOX14 Have Unique Gene Targets

**Figure 9:**
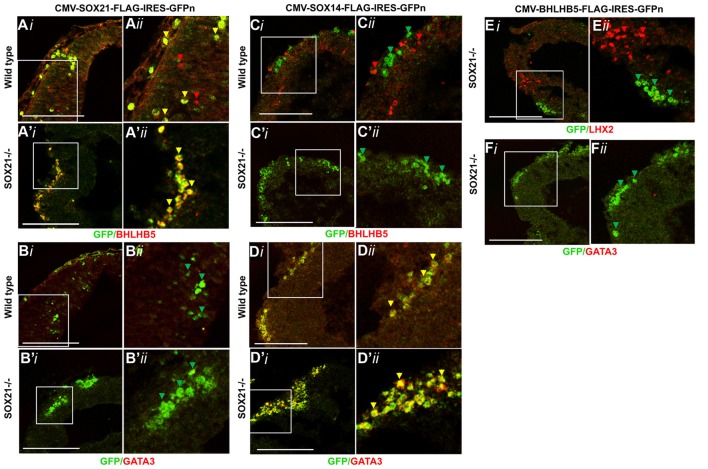
Overexpression of SOX21, SOX14 and BHLHB5 through *in utero* electroporation. Wild type embryos (*i,ii*) were *in utero* electroporated at E11 with Plasmids encoding CMV-SOX21-FLAG-IRES-GFP^n^, CMV-SOX14-FLAG-IRES-GFP^n^ and CMV-BHLHB5-IRES-GFP^n^. The immunostaining of the embryos with BHLHB5 illustrated that ectopic expression of this gene could be achieved when SOX21 was overexpressed **(A)** and not with SOX14 **(C)**. Expression of GATA3 on the other hand was only induced when SOX14 was overexpressed **(D)** and not with SOX21 **(B)**. These experiments were repeated on SOX21^−/−^ embryos (*iii,iv*) providing similar findings **(A’,B’,C’,D′)**. Interestingly, ectopic expression of BHLHB5 was unable to induce either the GABAergic or Glutamatergic lineage as show by staining of the embryos with GATA3 or LHX2 **(E,F)**. Scale bar, 75 μm.

Based on the previous findings, we investigated whether overexpression of SOX21 is sufficient for the induction of BHLHB5 through *in utero* electroporation. In our experiments, we observed good viability (70%) and good gene transfer (60% had more than 20 GFP^+ve^ cells per section) when embryos at E12.5 and later stages were electroporated with five pulses of 30–35 mV (50 ms-on and 950 ms-off). Embryos at E11 had low viability when electroporated at 30–35 V (22.2%) but higher viability (73.3%) and modest gene transfer (65% had 5–20 GFP^+ve^ cells per section) when electroporated with five pulses of 25 mV (50 ms-on and 950 ms-off; Supplementary Figure S5). Despite the lower efficiency at E11, we selected this time point since there is little to no expression of the endogenous BHLHB5 and GATA3 in the dorsal midbrain. Overexpression of SOX21 was indeed able to induce the expression of BHLHB5 in wild type embryos as well as rescue its expression in the SOX21 knockout mice (Figures 9A,A′). Interestingly, overexpression of SOX21 did not induce ectopic expression of GATA3 (Figures 9B,B′). We further wanted to verify whether SOX21 acts as a transcriptional repressor for the induction of BHLHB5 using a fused SOX21 HMG box to the repressor domain of Engrailed (EnR) and a fused SOX21 HMG box to the activator domain of Vp16. Indeed, the expression of BHLHB5 was only induced when SOX21-HMG-EnR was electroporated and not with SOX21-HMG-VP16 (Supplementary Figures S6A,B). Taking into consideration previous studies, we wanted to examine whether the induction of BHLHB5 was due to the antagonistic interaction of SOX21 and the SOXB1 proteins by electroporating a fused SOX1 HMG box to the repressor domain of Engrailed. Interestingly, no ectopic expression of BHLHB5 both in the wild type and SOX21 knockout embryos was observed; demonstrating that SOX21 can act independently of the SOXB1 genes (Supplementary Figure S6C).

The SOXB2 genes SOX21 and SOX14 are closely related and are believed to have the same gene targets, consequently we tested if SOX14 can induce BHLHB5 when ectopically expressed though *in utero* electroporation at E11. Curiously, overexpression of SOX14 was unable to induce the expression of BHLHB5 in wild type embryos (Figure 9C). Subsequently we overexpressed SOX14 in embryos lacking SOX21 to evaluate whether the failure to induce BHLHB5 was due to the presence of endogenous SOX21 occupying the same targets. In both cases SOX14 failed to induce BHLHB5 demonstrating that SOX21 has a unique role in the expression of this factor (Figures 9C,C′). Despite the failure to induce the expression of BHLHB5, overexpression of SOX14 promoted the expression of GATA3 illustrating that SOX14 is both necessary and sufficient to induce the GABAergic lineage (Figures 9D,D′).

Finally, while the reduction of BHLHB5 in the lentiviral knock down experiments did not affect the expression of GATA3 or LHX2, we wanted to test whether overexpression of BHLHB5 could induce their expression. Interestingly, overexpression of BHLHB5 through *in utero* electroporation at E11 was unable to induce the ectopic expression of either LHX2 or GATA3 (Figures 9E,F).

## Discussion

Despite the biological significance of the dorsal midbrain, the mechanisms regulating its development remain largely unknown since the process of neurogenesis has mostly been studied in the cerebral cortex and spinal cord. Previous studies illustrated that SOXB1 members and the SOXB2 transcription factor *SOX21* are highly expressed in undifferentiated cells of the VZ throughout the developing CNS (Uchikawa et al., 1999; Sandberg et al., 2005; Bani-Yaghoub et al., 2006; Wang et al., 2006; Cunningham et al., 2008). In contrast, the SOXB2 member *SOX14* has been previously detected in a subpopulation of midbrain, hindbrain and spinal cord differentiated cells (Wegner, 2011; Delogu et al., 2012; Popovic et al., 2014). Furthermore, SOX14 has been shown to affect the migration of specific ventral lateral geniculate nucleus GABAergic neurons which originate from the dorsal midbrain (Delogu et al., 2012). Nevertheless, there is no extensive data characterizing the expression of the *SOXB2* genes in the developing midbrain, therefore their dynamic expression was initially mapped at embryonic days E10.5, E11.5, E12.5 and E13.5. Indeed, in our study the expression of SOX21 was detected in the VZ progenitors and SVZ throughout the development of the dorsal midbrain (Figures 1A–F) and in a subset of GABAergic cells at the MZ following E11.5 (Figures 2D,L). At the early stages of development, it was demonstrated that *SOX14*^+ve^ cells emerge from the M3 and M4 domains of the midbrain and at later stages this population of cells expands along the dorsal midbrain (Figures 1G–L). Unlike SOX21, SOX14 was shown to be expressed in all of the GABAergic neurons of the dorsal midbrain at E12.5 (Figures 2J–L).

The pioneering experiments provided by Muhr and his group illustrate that the process of neurogenesis requires the parallel activity of Notch signaling and the expression of SOXB transcription factors where the levels of *SOX21* and the *SOXB1* genes regulate the progenitor vs. commitment determination (Sandberg et al., 2005). Despite the model proposed by Muhr and his colleagues, the deletion of *SOX21* did not affect the process of neurogenesis here since no abnormalities in the proliferation of the VZ progenitors were detected (Figures 3B,C,E). Furthermore, in our study we observed a dramatic reduction of BHLHB5 (Figures 4D,E) and GATA3 (Figures 5A,E) in the absence of SOX21, while the expression of the lineage-determining factors MASH1/GATA2 and DBX1 were unaffected (Figure 4). These findings indicate that SOX21 has a role in the commitment of certain neuronal precursors in the SVZ following their initial lineage determination. It is important to consider that previous studies in the spinal cord indicated that despite the co-localization of BHLHB5 with GATA2 at the P2 domain, BHLHB5 inhibits the expression of GATA3 in the V2a glutamatergic interneurons (Skaggs et al., 2011). In contrast, BHLHB5 has been shown to be an essential factor for the differentiation of a subset of inhibitory interneurons in the dorsal horn (Ross et al., 2012). Therefore, in this study we wanted to elucidate the role of BHLHB5 in the dorsal midbrain development to determine whether the loss of GATA3 could be attributed to defects of the BHLHB5^+ve^ precursors. Interestingly, in our gain of function experiments BHLHB5 failed to ectopically induce either LHX2 or GATA3 expression (Figures 9E,F); perhaps due to the lack of its partner proteins. Moreover, lentiviral knock down of BHLHB5 could not replicate the reduction of GATA3 (Figures 8A*ii*,E*ii*,F) as seen in the SOX21 knock out (Figures 5A,E) despite the efficient reduction of BHLHB5 (Figure 8B*ii*). It is also important to consider that reduction of BHLHB5 did not affect the expression of LHX2 expression (Figure 8E*ii*). Therefore we conclude that the loss of GATA3 in the SOX21 knock out embryos can be attributed to the direct role of SOX21. Indeed, while SOX21 could not induce the ectopic expression of GATA3 in the gain of function experiments (Figures 9B,B′), GATA3^eGFP^ embryos which lacked SOX21, expressed a severe reduction of the endogenous GATA3; despite the numbers of the GFP^+ve^ cells were unaffected (Figure 6A). These findings suggest that SOX21 has a selective role in maintaining the expression of GATA3 in the GABAergic precursors for their proper differentiation. GATA2 and GATA3 have been shown to promote GABAergic differentiation during neural development; however, in our study despite the severe loss of GATA3 in the SOX21 knockout embryos, the expression of the GABAergic terminal differentiation marker GAD6 was unaffected (Figures 5C,E). This result is in agreement with previous studies using GATA3 knockout embryos, suggesting that this factor is redundant in the process of GABAergic neuron differentiation during the early development of the ventral midbrain (Achim et al., 2014). Nevertheless, a small reduction of GFP in the axons of the GATA3^eGFP^ dorsal midbrain GABAergic axons was observed in the absence of SOX21 (Figures 6A–C). This indicated a possible defect in the maturation of the dorsal midbrain GABAergic neurons in the absence of SOX21 as exhibited by the clarified E12.5 brains from GATA3^eGFP^/SOX21^−/−^ mice which were used for a detailed observation of their axonal growth. Curiously, mice lacking SOX21 had shorter axons in comparison to wild type embryos suggesting that despite the absence of obvious defects in the terminal differentiation of GABAergic neurons, SOX21 and GATA3 are required for their proper axonal development and maturation (Figure 6D).

Since SOX14 is expressed in all the GABAergic neurons of the dorsal midbrain we speculated that it may compensate for the loss of GATA3 and SOX21. The loss of SOX14, unlike SOX21, caused a severe reduction in GATA3, GABA and GAD6 (Figures 7B–D,G) as well appearing to promote ectopic expression of GATA3 in the gain of function experiments (Figure 9D). These data indicate that SOX14 is both necessary and sufficient to promote the terminal differentiation of the dorsal midbrain GABAergic neurons while SOX21 maintains their identity for their proper maturation. Interestingly, TAL2 was reduced in the SOX14 knock out embryos despite SOX14 being exclusively expressed in the post mitotic GABAergic neurons (Figure 7G). Taking into consideration previous studies, we speculate that SOX14 could be responsible for maintaining the expression of TAL2 and promoting the expression of GATA3 and GAD6 in the GABAergic neurons after they exit the SVZ (Achim et al., 2013).

**Figure 10:**
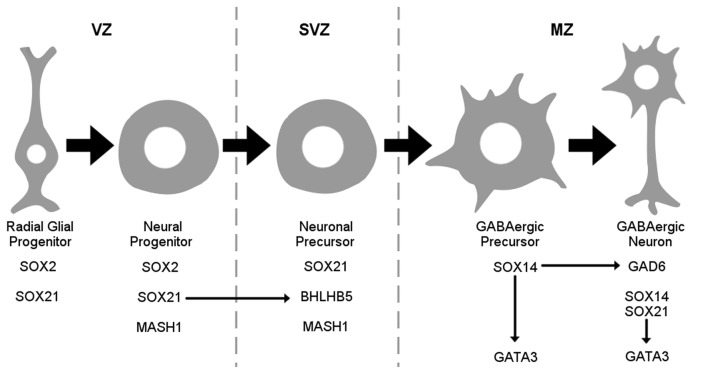
Schematic representation of the SOX2B during maturation of GABAergic neurons. SOX21 is expressed both in radial glial and neuronal progenitors at the VZ while been maintained in neuronal precursors at the sub ventricular zone (SVZ) where it appears to govern the expression of BHLHB5. At the MZ SOX14 marks the GABAergic lineage while promoting GATA3 expression.

It is important to consider that the SOXB2 genes are paralogs and are believed to have the same gene targets due to their homology. Therefore, their differential role is speculated to be due to their temporal and spatial expression. However, in our study we provide evidence that these factors instead have unique gene targets through our gain of function experiments. Unlike SOX21, SOX14 was unable to induce the expression of BHLHB5 in both wild type and SOX21 knockout embryos while SOX21 was unable to induce the ectopic expression of GATA3 (Figures 9A–D). This is illustrated in Figure 10 where the differential expression of SOX14 and SOX21 is exemplified. Even though our work has concentrated on the dorsal midbrain we have collected some data relating to the ventral midbrain GABAergic neurons where both SOX14 and SOX21 are expressed (data not shown; Achim et al., 2013). We have found that loss of either SOX14 or SOX21 does not seem as detrimental to the GABAergic fate as in the dorsal midbrain. Similarly, deletion of the GABAergic fate determining factor MASH1 resulted in the severe reduction of GABAergic neurons in the dorsal midbrain but not in the ventral region (Peltopuro et al., 2010). Perhaps, this effect is indicative of a more heterogeneous GABAergic neuron population found in the ventral midbrain. Taken together, our data provide new insight into the role of SOXB2 factors and do not support a pan-neurogenic function, but instead lend credence to a model whereby the differentiation of GABAergic lineages in the dorsal midbrain is likely though the sequential regulation of distinct downstream target genes.

## Author Contributions

Acquisition of data and experimentation was conducted by NM while the Lentiviral production was conducted by PF. CK and GL were responsible of the production, maintenance and husbandry of the mouse models. Analysis and interpretation of data was performed by NM and EP. Drafting of manuscript and critical revision was carried out by EP and SM. All authors read and approved the final manuscript.

## Conflict of Interest Statement

The authors declare that the research was conducted in the absence of any commercial or financial relationships that could be construed as a potential conflict of interest.
