# Supplementary material for: Sequential Role of SOXB2 Factors in GABAergic Neuron Specification of the Dorsal Midbrain
Source: Front Mol Neurosci. 2018 May 8;11:152. doi: 10.3389/fnmol.2018.00152 (PMC5952183; doi:10.3389/fnmol.2018.00152)
Supplement: Supplementary file 1 [file Data_Sheet_1.pdf]

Supplementary Data

Tables:

Table S1. Oligos used for the genotyping of the mice, RT-Q-PCR & plasmid vectors

Genotype	5' sequence (5' to 3')	3' sequence (5' to 3')
Sox21^{WT}	CTCTGGTACCCACTAGTAACGCTGCTCAAAAAGGACAAG	GGATCCTTAATTAATTACTTCTCGGGGTTCGCGAGCAG
Sox21^{KO}	ATCCATATGGCTAGCGCTACCG	GGGCAGCTTGCCGGTGGTGC
Sox14^{WT}	AGCGGGCATGACTTCTGCGC	GCTCCTCGCCCTTGCTGCTCACC
GAPDH Q-PCR	CACATCGCTCAGACACCATG	ATGGTTCACACCCATGACGA
MASH1 Q-PCR	CAGCCCCGCTTCCTTCAAGG	AAGTCCATTCCCAGGAGAGC
DBX1 Q-PCR	GAGCCAGGTCCTGTCAAACA	GGGAGACGGTGCCCTTATTC
NESTIN Q-PCR	CTCGGGAGAGTCGCTTAGAG	GGGAGCCTCAGACATAGGTG
NHLH1 Q-PCR	ACCTGAGAGTTGGAGATGGGA	ATGCTTTGTGCTTAAGTGCCC
TAL2 Q-PCR	GCGTTTGATGGGAGGCAAAT	AAAGGAGGCTTTCCACACACA
NGN2 Q-PCR	TGTAGGATCCATGTTCGTCAAATCTG	ATTGAGAATTCGATACAGTCCCTGG
NEUROD1 Q-PCR	TTATTGCGTTGCCTTAGCACT	TGAGTGTTATGGGTCTGGTTTCT
GATA3 Q-PCR	AACTGCAGCTCCTTCAACCCGGCC	AAGAATTCTGACAGTGTGCCCATTTGG
LXH2 Q-PCR	GTTGGCTGAGAGCTTCCGTA	ATCCGAACAGACTTCCACCTT
SOX21 Q-PCR	GCTAGCGAATTCGTATAGGTGTCAGGCAGAGG	AGATCCGGGCTGTGTTCTGC
SOX14 Q-PCR	AATCCTGAGCTATCAAGCCG	TCTCCCGGGAGGAGGACAGG
GAD6 Q-PCR	CCTACTGCAGTGTCCTAGGGACCC	GGCTACTGAAGCAAGATTTCC
BHLHB5 Q-PCR	TGCTCCCCATTCCTTTTCCTT	AAGGCTGAATGTCCGGTTTGT
BHLHB5sh	CCGGGCCAACAAATATGAAGAGAAACTCGAGTTTCTCTTCATATT TGTTGGCTTTTTG	AATTCAAAAAGCCAACAAATATGAAGAGAAACTCGAGTTTCTCT TCATATTTGTTGGC
SOX21sh	CCGGCCGGTTTGTATGTACATAGATCTCGAGATCTATGTACATAC AAACCGGTTTTTG	AATTCAAAAACCGGTTTGTATGTACATAGATCTCGAGATCTATG TACATACAAACCGG
SOX14sh	CCGGAGCGGCCTTACATCGATGAAGCTCGAGCTTCATCGATGTAA GGCCGCTTTTTG	AATTCAAAAAAGCGGCCTTACATCGATGAAGCTCGAGCTTCATC GATGTAAGGCCGCT

Figures:

Fig. S1

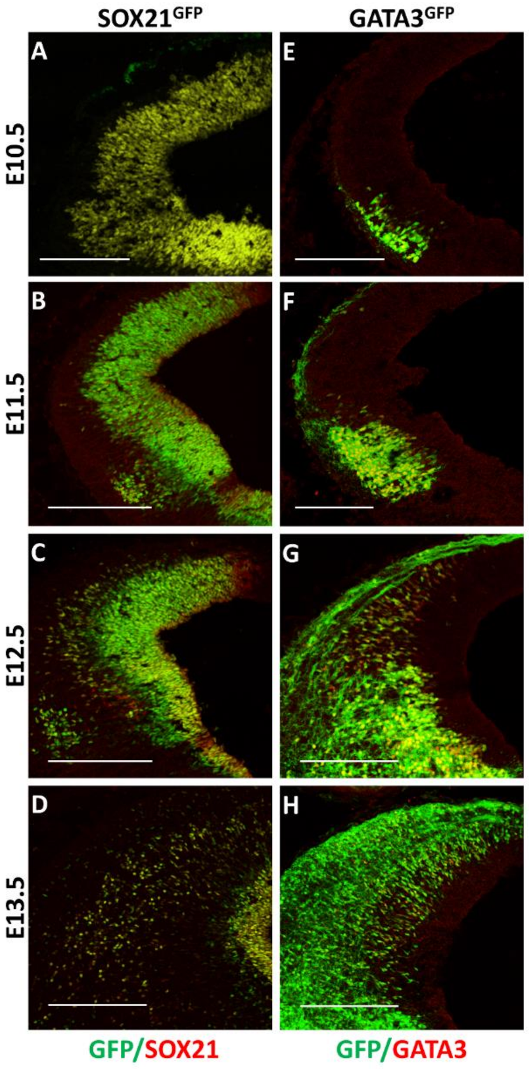

Fig. S1. Validation of the SOX21^{GFPn} and GATA3^{GFPn} reporter lines. Midbrain coronal sections of *SOX21*^{GFPn} and GATA3^{GFPn} mouse embryos were stained at different stages of early development with SOX21 and GATA3 antibody respectively (A & E-E10.5, B & F-11.5, C & G-E12.5 & D & H-E13.5). Immunostaining of the sections demonstrated a complete co-localization of the GFP with the respective antibody in the dorsal midbrain. Scale bar, 150µm.

Fig. S2

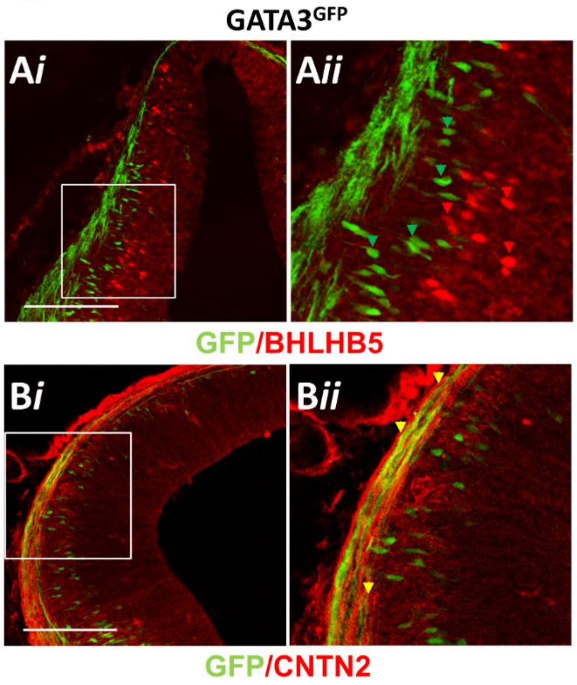

Fig. S2. Characterisation of GATA3^{eGFP} dorsal midbrain sections at E12.5 in relation to BHLHB5 and CNTN2. Coronal sections of *GATA3^{GFPn}* midbrains stained with BHLHB5 (Ai, Aii) and CNTN2 (Bi, Bii) antibodies. Red arrows indicate BHLHB5^{+ve} cells, green arrows indicate GATA3^{+ve} cells and yellow arrows denote co-localisation between CNTN2 and the GFP^{+ve} axons. Scale bar, 150µm.

Fig. S3

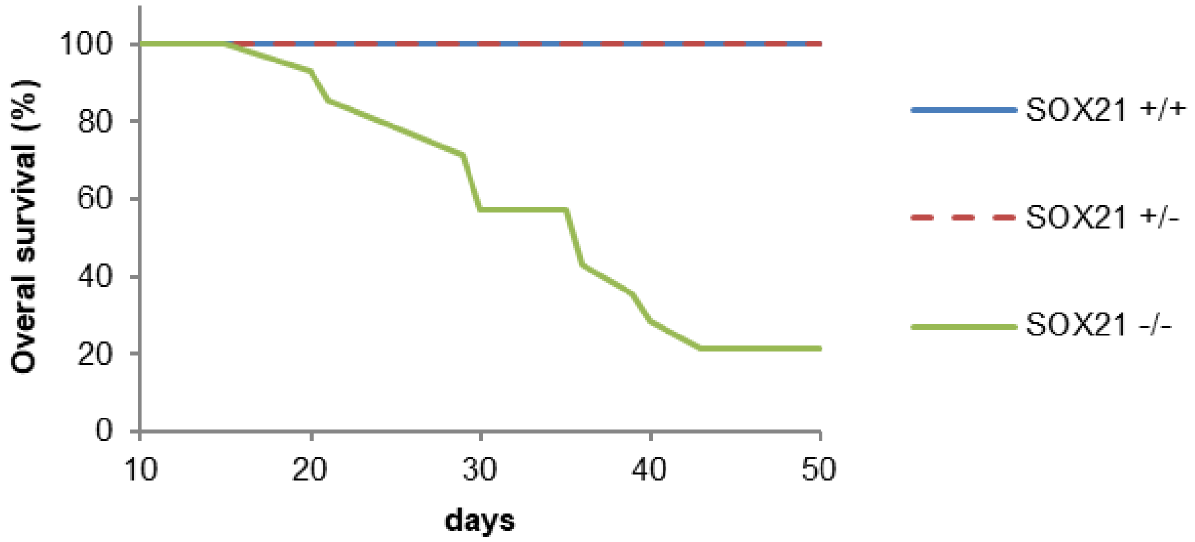

Fig. S3. Kaplan Meier survival curve of SOX21$^{-/-}$. Mice lacking SOX21 exhibited a significant 70% lethality in comparison the wild type and heterozygous mice (n=31, P=0.0002, DF=1, 95% CI=31.546-91.348, comparison of proportions).

Fig. S4

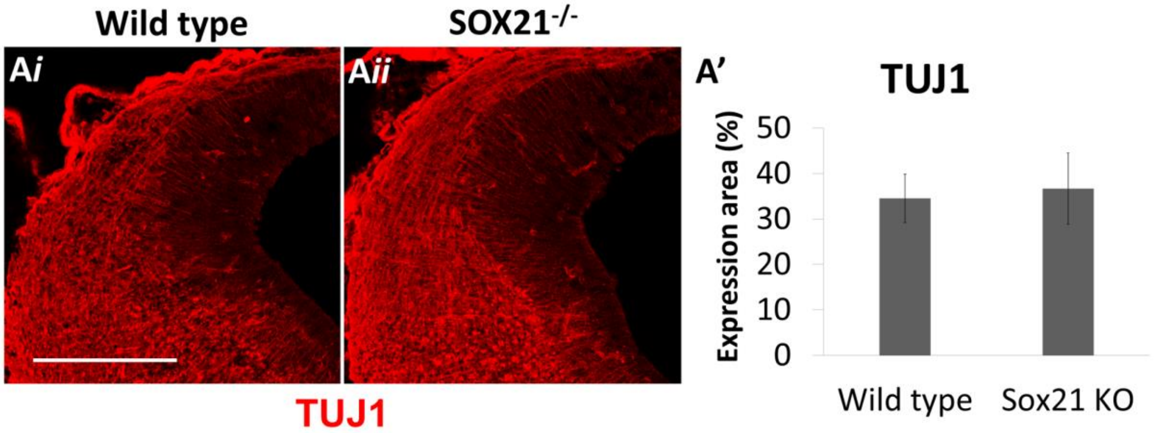

Fig. 4. Expression of TUJ1 in SOX21$^{-/-}$ and wild type embryos at E12.5. The immunostaining of the post-mitotic neuronal marker TUJ1 (A & A') was similar between knockout (*ii*) and wild type embryos (*i*). Data are represented as mean±s.e.m, n=8 (4 pairs of mice). Scale bar, 150µm.

Fig. S5

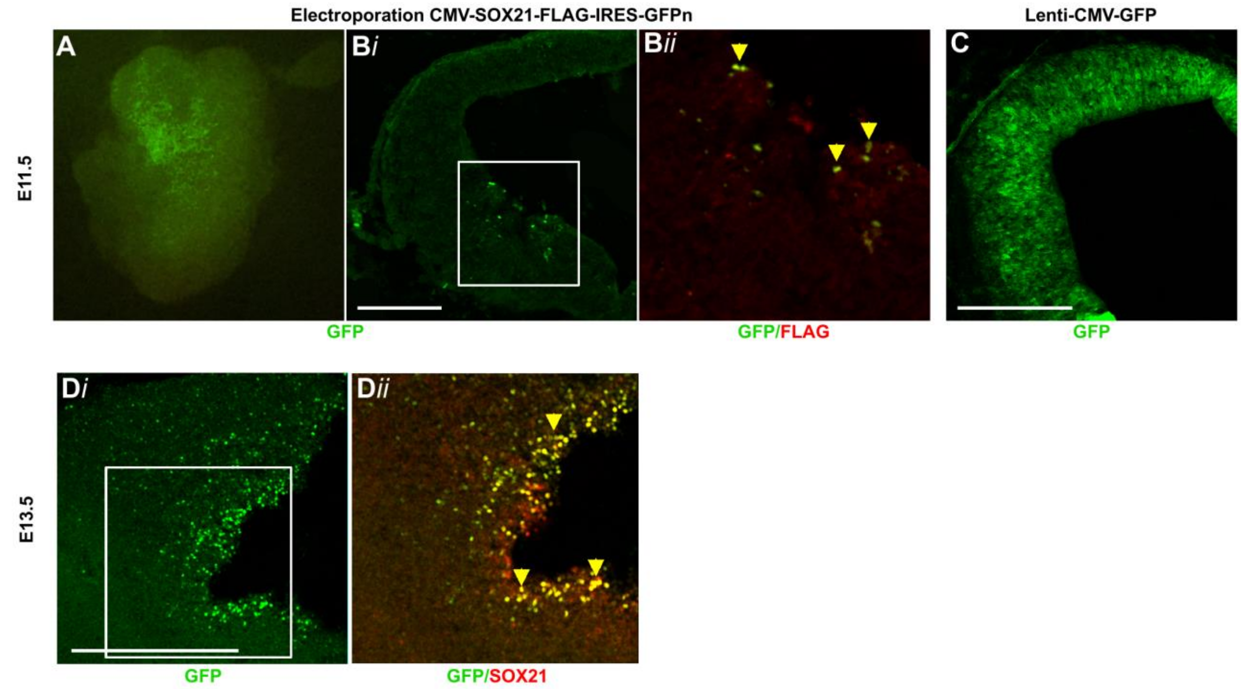

Fig. S5. Validation of the plasmid delivery. E11 embryos were electroporated with a CMV-SOX21-FLAG-IRES-GFPn plasmid. The embryos were checked under the UV to verify successful delivery (A) and the expression of the vector was validated with immunostaining of the FLAG tag (B*i*-*ii*). Electroporation of the CMV-SOX21-FLAG-IRES-GFPn plasmid at E12.5 had a modest gene transfer and expression of SOX21 (D*i*-*ii*). Embryos infected at E8.5 with a lentiviral CMV-GFP vector through intra-amniotic injections was the most successful delivery method as the embryos expressed the highest amount of GFP (C). Scale bar, 150µm.

Fig. S6

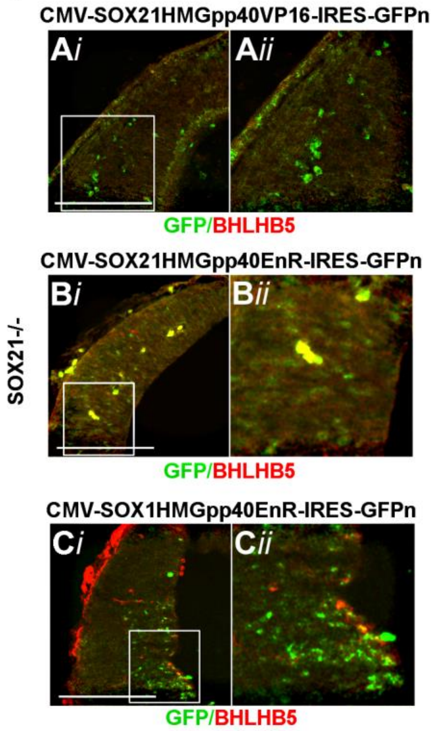

Fig. S6. In utero electroporation of SOX21HMGVP16, SOX21HMGEnR and SOX1HMGEnR plasmids. SOX21$^{-/-}$ embryos were in utero electroporated at E11 with Plasmids encoding CMV-SOX21HMGpp40EnR-IRES-GFP^{n}, CMV-SOX21HMGpp40VP16-IRES-GFP^{n} and CMV-SOX1HMGpp40EnR-IRES-GFP^{n}. The immunostaining of the embryos with BHLHB5 illustrated that ectopic expression of this gene could be only achieved when CMV-SOX21HMGpp40EnR-IRES-GFP^{n} was electroporated (B*i*-*ii*) and not with the other two fusion protein (A*i*-*ii* & C*i*-*ii*). Scale bar, 75µm.
